# Systems, Subjects, Sessions: To What Extent Do These Factors Influence EEG Data?

**DOI:** 10.3389/fnhum.2017.00150

**Published:** 2017-03-30

**Authors:** Andrew Melnik, Petr Legkov, Krzysztof Izdebski, Silke M. Kärcher, W. David Hairston, Daniel P. Ferris, Peter König

**Affiliations:** ^1^Institute of Cognitive Science, University of OsnabrückOsnabrück, Germany; ^2^Human Research and Engineering Directorate, U.S. Army Research LaboratoryAdelphi, MD, USA; ^3^School of Kinesiology, University of MichiganAnn Arbor, MI, USA; ^4^Department of Neurophysiology and Pathophysiology, University Medical Center Hamburg-EppendorfHamburg, Germany

**Keywords:** comparison of EEG systems, ANT Neuro asalab, Brain Products actiCAP, g.tec g.Nautilus g.SAHARA dry electrodes, Emotiv EPOC, auditory evoked potential AEP N1 P2, steady-state visually evoked potential SSVEP, face sensitive N170

## Abstract

Lab-based electroencephalography (EEG) techniques have matured over decades of research and can produce high-quality scientific data. It is often assumed that the specific choice of EEG system has limited impact on the data and does not add variance to the results. However, many low cost and mobile EEG systems are now available, and there is some doubt as to the how EEG data vary across these newer systems. We sought to determine how variance across systems compares to variance across subjects or repeated sessions. We tested four EEG systems: two standard research-grade systems, one system designed for mobile use with dry electrodes, and an affordable mobile system with a lower channel count. We recorded four subjects three times with each of the four EEG systems. This setup allowed us to assess the influence of all three factors on the variance of data. Subjects performed a battery of six short standard EEG paradigms based on event-related potentials (ERPs) and steady-state visually evoked potential (SSVEP). Results demonstrated that subjects account for 32% of the variance, systems for 9% of the variance, and repeated sessions for each subject-system combination for 1% of the variance. In most lab-based EEG research, the number of subjects per study typically ranges from 10 to 20, and error of uncertainty in estimates of the mean (like ERP) will improve by the square root of the number of subjects. As a result, the variance due to EEG system (9%) is of the same order of magnitude as variance due to subjects (32%/sqrt(16) = 8%) with a pool of 16 subjects. The two standard research-grade EEG systems had no significantly different means from each other across all paradigms. However, the two other EEG systems demonstrated different mean values from one or both of the two standard research-grade EEG systems in at least half of the paradigms. In addition to providing specific estimates of the variability across EEG systems, subjects, and repeated sessions, we also propose a benchmark to evaluate new mobile EEG systems by means of ERP responses.

## Introduction

Electroencephalography (EEG) techniques have matured over the last 80-plus years since the study of the electrical activity of the human brain by Berger (1935). The hardware and processing methods for EEG data have advanced to being very high quality. Recording scalp potentials in fixed temporal relation to events, e.g., stimulus presentation or reports of recognition by button presses, allows for the capture of electrocortical activity related to sensory, motor, or cognitive processes. Such scalp potentials recorded and averaged over many trials, called event-related potential (ERP; Luck, 2005; Sur and Sinha, 2009), help to reveal important insights about how the human brain works. For example, processing of auditory stimuli was studied with the N100 ERP component (Davis, 1939) and the N170 ERP component relates to the neural processing of faces (Rossion and Caharel, 2011). Currently, EEG is one of the most widely used techniques in noninvasive brain research to study correlates of perceptual, cognitive, and motor activity associated with processing of information.

A shift in the field of neuroscience to a greater appreciation of natural behavior is placing new demands on recording techniques. Participants have to act and move in order to experience the proprioceptive and vestibular sensations under natural conditions (McDowell et al., 2013; Snider et al., 2013; Gramann et al., 2014), parallel to a simultaneous recording of participants’ motor actions and external events influencing cognition. Many traditional noninvasive imaging techniques to estimate brain activity, like functional magnetic resonance imaging (fMRI; Huettel et al., 2004) or magnetoencephalography (MEG; Hansen et al., 2010), have mobility constraints that limit the ability for use in natural conditions. In contrast, mobile brain/body imaging (“MoBI”) has the potential to provide unique insight into cognition in normal everyday behaviors (Ojeda et al., 2014). Near-infrared spectroscopy (NIRS; Workman and Weyer, 2007) allows mobility and can provide blood oxygenation level dependent signals regarding brain function, but its temporal resolution is not close to that of EEG. The high temporal resolution of EEG makes it the prime candidate for a measurement technique of brain dynamics in modern real-world paradigms investigating cognition under natural contexts and those involving sensorimotor coupling and actions (Makeig et al., 2009; Gramann et al., 2010; De Sanctis et al., 2014; Aspinall et al., 2015).

A desire for making EEG recordings under natural conditions, and potential uses in the gaming and wellness industries, have triggered the development of affordable and mobile EEG systems. These systems tend to be low-budget, easy to set up, and convenient for wearing over an extended period of time (Hairston et al., 2014; Hairston and Lawhern, 2015). Multiple aspects of EEG systems can influence the quality of the signal. For example, conventional EEG sensors are usually based on a conductive gel, which leads to time-consuming setup and gel removal from the hair and the electrodes afterward. Newer systems can use dry electrodes or saline-based electrodes. EEG amplifiers can have varying parameters like noise performance, power consumption, signal bandwidth, and cost (Badillo et al., 2003; Harrison and Charles, 2003; Lin et al., 2006; Hairston et al., 2015). More recent developments include wireless data transition directly from a cap (De Vos et al., 2014), ultra-low power digitization, and a minimal use of cables (Warchall et al., 2016). All of these aspects have a potential to alter EEG data. How much do these changes affect the recorded signals? Are the results obtained with new mobile EEG systems and research-grade EEG systems equivalent?

Recent studies have attempted to compare recordings obtained from different EEG systems. In one study (Gargiulo et al., 2010), investigators compared a new EEG system with dry electrodes to a standard and clinically available EEG system with wet electrodes using parallel and serial recording methods. The comparison included two experimental paradigms, in which frequency domain and correlation coefficients of channel data, and ERPs were compared. Another study examined the correlation of signals from dry foam-based EEG sensors and wet EEG sensors, as well as the impedance at the sensor-skin contact during long-term EEG measurements (Liao et al., 2012). Yeung et al. (2015) completed a comparison of foam-based and spring-loaded dry EEG electrodes using three experimental paradigms by linear correlation analyses. Additional studies have looked at the application-based performance using P300 brain-computer interfaces with dry and gel-based electrodes (Guger et al., 2012; De Vos et al., 2014). The general conclusion from these studies was that EEG data could be successfully collected using non-research grade EEG systems when taking into account the number and placement of electrodes.

With the increasing number of comparisons of EEG systems, we need a quantitative standard for comparing EEG systems (Oliveira et al., 2016b). A systematic benchmark based on a variety of paradigms testing different brain-response effects would allow assessing how results vary across EEG systems (low-budget, dry electrode, research-grade). That will allow comparing not only systems evaluated in a particular article but also any new EEG system (Senevirathna et al., 2016) in the future to the database of performance results currently being collected. Furthermore, to rightly estimate how large variability due to systems is, we have to compare it to other factors of variance, like subjects or repeated sessions for each subject-system combination.

The primary purpose of this study was to quantify the variance across EEG systems, subjects, and sessions for some standard EEG measures. We tested four EEG systems: two standard research-grade systems, one system designed for mobile use with dry electrodes, and one mobile low-budget system with a lower channel count. We used six well-established ERP paradigms: (1) auditory evoked potentials (AEPs); (2) steady-state visually evoked potential (SSVEP); (3) motor potentials (MPs); (4) visual mismatch negativity (vMMN); (5) face-sensitive N170 component; and (6) target-distractor visual decision-making (vDM). We recorded four subjects three times with each of the four EEG systems. This setup allowed us to assess the influence of all three factors (System, Subject, Session) on the variance of EEG data.

## Materials and Methods

### General Methods

#### Subjects

Four paid healthy volunteers (three males, mean age: 24 years, range 23–25 years), participated in the study. All subjects had normal or corrected-to-normal vision (self-reported) and normal hearing (self-reported). Subjects sat in a darkened EEG recording chamber in front of a computer monitor from a distance of 90 cm and with a headset on. We obtained written informed consent from all subjects before the experiment and the protocol had been approved by the University Osnabrück ethics committee for protection of human subjects.

#### Design

**Figure 1:**
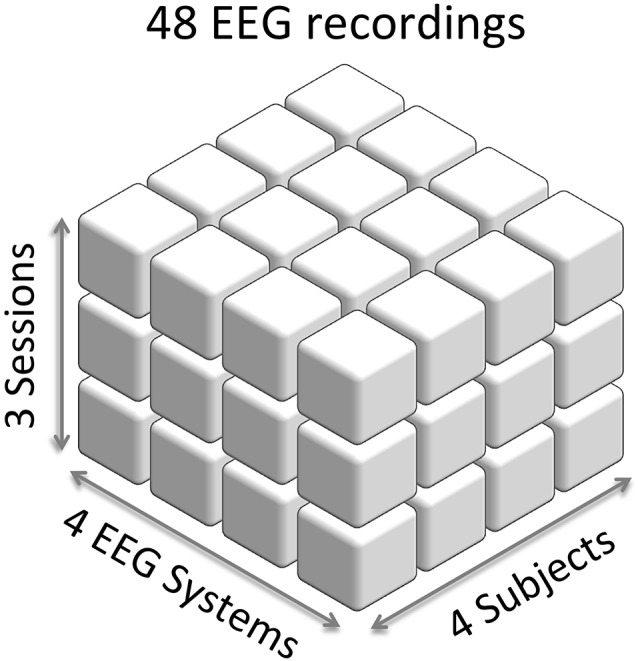
**Experimental setup of electroencephalography (EEG) recordings.** Each cube represents an EEG recording. We recorded four subjects with four EEG systems and three sessions for each combination of a subject and an EEG system. This results into 48 EEG recordings in the study.

**Figure 2:**
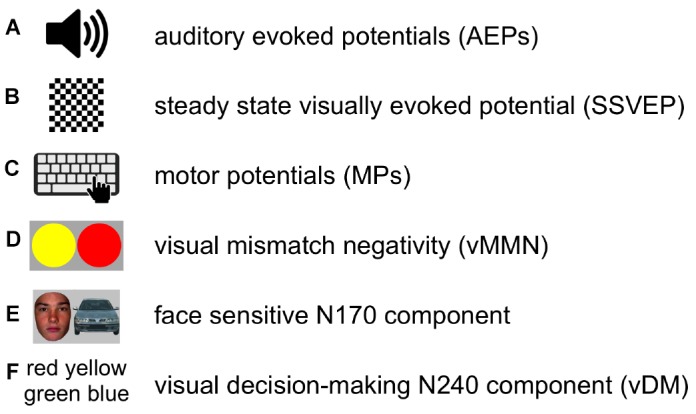
**In each recording session, subjects performed a battery of six standard EEG paradigms.** Pictograms **(A–F)** schematically represent the paradigms.

We recorded each subject three times with each of four EEG systems, which gave 12 EEG sessions per subject or 48 EEG sessions in the study (Figure 1). Median time interval between recording sessions was 2 day. In each recording session, subjects performed a battery of six standard EEG paradigms (Figure 2) based on ERPs and SSVEP: (Figure 2A) AEPs; (Figure 2B) SSVEP, evoked by an alternating contrast checkerboard; (Figure 2C) MPs elicited by voluntary tapping; (Figure 2D) vMMN (ERP waveforms subtraction “deviant minus standard”); (Figure 2E) face-sensitive N170 component (ERP waveforms subtraction “faces minus cars”); (Figure 2F) vDM N240 component (ERP waveforms subtraction “targets minus distractors”). The current selection of paradigms covers different modalities (visual, auditory, as wells as motor) and ranges from a low level to a higher level cognitive load. This setup allowed us to assess the influence of the factors: System, Subject, and Session on EEG data in different standard EEG paradigms.

The order of the paradigms was identical for all recordings and had the following sequence: vMMN, SSVEPs, N170, MPs, vDM, AEP. We had additional paradigms which related not to ERPs but to time-frequency analysis and eye movements, which are however beyond the scope of the present article. The additional paradigms took approximately 20 min before and 20 min after the paradigms processed in the current study. Each of the paradigms in the current study was divided into blocks and subjects could decide themselves when to start a new block and how long pause to take between blocks. Thus, duration of a recording session varied between 2 h and 3 h, depending on how long pauses between blocks and paradigms took a subject.

#### Paradigm 1: Auditory Evoked Potentials

In the auditory paradigm (Figure 2A), we presented short stimuli by a headset every 600 ms. The duration of the tone was 200 ms and interstimulus interval was 400 ms. Stimuli in the first half of the paradigm were pure tones at the frequency 1 kHz, and stimuli in the second half of the paradigm were white-noise audio signals. The paradigm required no response, but only to passively hear tones. Therefore we asked subjects to close their eyes. We assumed that it should help subjects to better concentrate on the task, relax their eyes, and avoid eye-blinks. One-thousand stimuli in the paradigm were equally distributed across four blocks. The peak-to-peak amplitude of the ERP components N1 and P2 defined the dependent variable in this paradigm.

#### Paradigm 2: Steady-State Visually Evoked Potential (SSVEP)

In this paradigm (Figure 2B) we presented an alternating contrast checkerboard on a monitor (visual angle: 3.5 × 3.5°) at the frequency of 12 Hz. Two-thousand and four-hundred alternations were equally distributed across four blocks. The paradigm required no response, but only to passively observe the flickering checkerboard. A 4 × 4-pixel cross in the center of the checkerboard served as an anchor for the fixation point. Minimum to maximum peak difference of the evoked potential defined the dependent variable in this paradigm.

#### Paradigm 3: Motor Potentials (MPs) Elicited by Voluntary Tapping

In this paradigm (Figure 2C) we asked subjects to rhythmically press the key Arrow Down on a keyboard with the index finger of the right hand with a constant pace of about once per second. At the start of each block, subjects heard a guiding pace through a headset, which consisted of 10 beeps played once per second. After that, subjects could start to press the key Arrow Down on the keyboard. We asked subjects to close their eyes. We assumed that it should help subjects to better concentrate on the task, relax their eyes, and avoid eye-blinks. The paradigm consisted of three blocks. In each block, subjects had to conduct 240 button presses. Thus we obtained 720 button presses in this paradigm. The peak-to-peak amplitude of the ERP components MP and Reafferent Potential (RAP) defined the dependent variable in this paradigm.

#### Paradigm 4: Visual Mismatch Negativity (vMMN)

In this paradigm (Figure 2D) we presented sequentially standard (red) and deviant (yellow) circles on a monitor (visual angle: 2 × 2°) with a deviant-to-standard stimuli ratio of 1–4. We spaced deviant stimuli according to Poisson distribution (Ord, 1967), while we set the minimal number of standard stimuli in between to 1 and the maximal number of standard stimuli in between to 8. We asked subjects to look and concentrate their attention at the blinking circles. The duration of a stimulus was equal to 280 ms. An interval between stimuli onsets was randomly selected for each new stimulus from intervals of 630 ms to 830 ms. One-thousand and two-hundred stimuli in the paradigm were equally distributed across four blocks. The difference-amplitude peak of subtracted ERPs (deviant stimuli ERP minus standard stimuli ERP) at around 200 ms after a stimulus onset defined the dependent variable in this paradigm.

#### Paradigm 5: Face-Sensitive N170 Component

In the paradigm (Figure 2E), we presented pictures of faces, cars and noise in a random sequence on a monitor (visual angle: 3.1 × 3.5°). Two sets of 43 colored photographs of full front faces (21 males) and cars were adopted from a different study (Rossion and Caharel, 2011). “Faces were presented without glasses, facial hair or make-up, and with neutral expression. All face pictures were trimmed to remove their variable backgrounds, clothing and hairline. Car pictures were also edited to remove background. Noise stimuli were made by scrambling the faces and the cars using a Fourier phase randomization procedure” (Rossion and Caharel, 2011). We asked subjects to look and concentrate their attention on the appearing pictures. The duration of a stimulus was equal to 280 ms. An interval between stimuli onsets was randomly selected for each new stimulus from intervals of 630 ms to 830 ms. One-thousand and thirty-two stimuli in the paradigm, with a ratio of face, car, and noise pictures equal to 1:1:1, were equally distributed across four blocks. The difference-amplitude peak of subtracted ERPs (face stimuli ERP minus car stimuli ERP) at around 170 ms after a stimulus onset defined the dependent variable in this paradigm.

#### Paradigm 6: Visual Decision-Making N240 Component (vDM)

In the paradigm (Figure 2F), we presented text labels with the German name of a color on a monitor (visual angle: 1.5 × 0.6°). We used four text labels: rot (red), grün (green), blau (blue), and gelb (yellow), with a ratio of 1:1:1:1. A sequence of the text labels was random, but no two identical color names in a row. The color of the font was always black and presented on a gray screen. We asked subjects to look and concentrate their attention at the appearing text labels and to press the key Arrow Down on the keyboard with the index finger of the right hand, only when the text label “rot” (red) appears on the monitor. Other text labels did not require any action. The duration of a stimulus was equal to 280 ms. The interval between stimuli onsets was 1 s long. In the case of an error response, subjects got feedback: a white flash of the full screen (duration of 150 ms) and extended interval to the next stimulus onset (2 s). Nine-hundred and sixty stimuli in the paradigm were equally distributed across four blocks. The difference-amplitude peak of subtracted ERPs (target stimuli ERP minus distractor stimuli ERP), at around 240 ms after a stimulus onset, defined the dependent variable in this paradigm.

### Physiological Methods

#### EEG Systems and Data Acquisition

**Figure 3:**
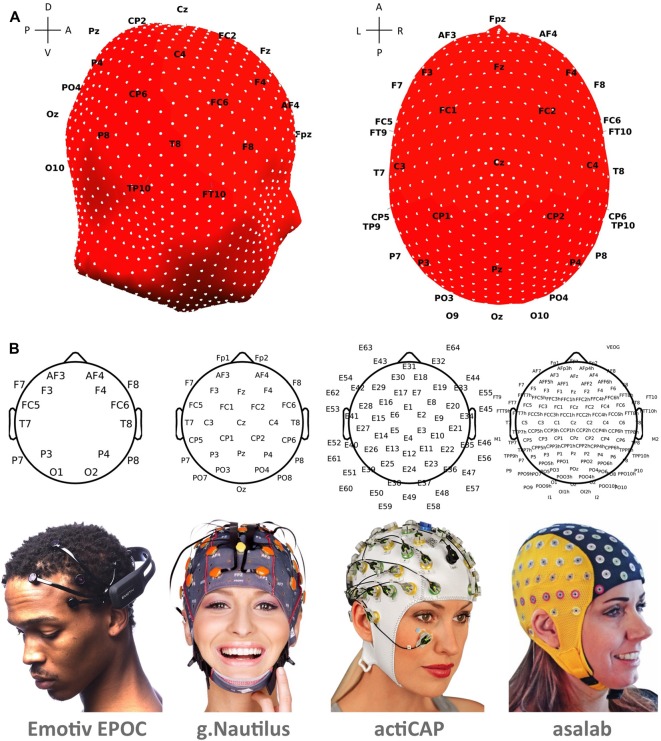
**(A)** Mesh-head model with the 1082 mesh channels shown as white dots. Black labels represent the 10–20 international system and depict positions of 35 electrodes. Labels at the crosses represent anterior (A), posterior (P), dorsal (D), ventral (V), left (L) and right (R) sides. **(B)** Allocation of electrodes. Beneath the topoplots are photos of the corresponding EEG systems used in the study (Photos with permission from Emotiv, g.tec, Brain Products, and ANT Neuro).

We recorded electroencephalographic (EEG) data using four EEG systems (Table 1 and Figure 3B): (1) asalab^TM^ (ANT Neuro, Enschede, Netherlands^1^); (2) actiCAP (Brain Products GmbH^2^); (3) g.tec’s g.Nautilus; and (4) Emotiv EPOC. We conducted EEG recordings in batches: first, we conducted a batch of recordings for all subjects with g.Nautilus, then with actiCAP, then with asalab^TM^, and the last batch of recordings with Emotiv.

**Table 1:** **General properties of the electroencephalography (EEG) systems**.

System	Manufacturer	Electrode type and material	Reference location	Ground location	Number of data channels	Sampling rate (Hz)	Wireless transmission
asalab^TM^	ANT Neuro	Gel-based Ag/AgCl	Cz	Left clavicle area	127	1024	None
actiCAP	Brain Products	Active gel-based Ag/AgCl	Cz	Near Fz	64	1000	None
g.Nautilus	g.tec	Active dry gold-alloy coated (g.SAHARA)	Right earlobe	AFz	32	500	2.4 GHz band
EPOC	Emotiv	Saline-infused felt	P3 (or mastoid)	P4 (or mastoid)	14	128 (2048 internal)	Bluetooth Smart 2.4 GHz

asalab^TM^ had 127 data channels and gel-based Ag/AgCl electrodes (waveguard^TM^ caps). Electrodes were positioned according to the 10-5 international system (Oostenveld and Praamstra, 2001). The recording reference was Cz and the ground electrode laid on the left clavicle area. We kept scalp impedances below 10 kΩ. The sampling rate of continuously recorded EEG data was 1024 Hz. Neither band-pass nor notch filter was applied. The system transmits EEG data via a shielded electrical cord.

actiCAP had 64 data channels and gel-based Ag/AgCl active electrodes. Electrodes were positioned according to the equidistant spherical montage. The recording reference was Cz and the ground electrode laid near Fz. We kept scalp impedances below 10 kΩ. The sampling rate of continuously recorded EEG data was 1000 Hz. Neither band-pass nor notch filter was applied. The system transmits EEG data via an electrical cord.

g.Nautilus had 32 data channels with g.SAHARA dry electrode technology and wireless data transmission. A receiver situated in proximity to the EEG system. Electrodes were positioned according to the 10-20 international system. The recording reference was on the right ear and the ground electrode AFz. The EEG system had dry active electrodes and high impedance amplifiers. Internal impedance check was performed automatically via software. The sampling rate of continuously recorded EEG data was 500 Hz. Neither band-pass nor notch filter was applied. g.Nautilus wirelessly transmits data via the 2.4 GHz band (Bluetooth). We shifted all timestamps (event triggers) in recordings with g.Nautilus for 11 ms earlier, since we encountered the constant delay in comparison to asalab^TM^ and actiCAP research-grade EEG systems.

Emotiv EPOC had 14 data channels, saline-based electrodes, and wireless data transmission. A receiver situated in proximity to the EEG system. Electrodes were positioned according to the 10-20 international system. The recording references in the CMS/DRL noise cancellation configuration were P3/P4 electrodes. Sensors were adjusted until connectivity reached the “green” level, indicating that the impedance level required by the software was reached (the Emotiv EPOC software development kit was used). The sampling rate of continuously recorded EEG data was 128 Hz. Emotiv EPOC has a built-in band-pass filter of 0.2–43 Hz and notch filters at 50 Hz and 60 Hz and wirelessly transmits data via the 2.4 GHz band (Bluetooth).

We noticed an issue of timestamps of event triggers with the Emotiv EPOC EEG system. Timestamps stored in the EEG data were highly susceptible to jitter and delay (Hairston et al., 2014). To overcome this issue we synchronized biased timestamps of the event triggers in EEG data with true timestamps of the same event trigger logged by a presentation script during the recordings. Precise synchronization at different recording devises is an important aspect because residual jitter might contribute to a reduction of ERP amplitude, specifically of high frequency.

Raw EEG data from all systems were band-pass filtered from 1 Hz to 43 Hz (Matlab function firfilt.m) to remove drift and to match the Emotiv EPOC’s built-in filter. Then we applied the standardized preprocessing PREP pipeline (Bigdely-Shamlo et al., 2015), which removed line-noise, robust referenced the signal, and interpolated bad channels. “The robust average reference procedure tries to estimate the true average of the EEG channels after removing contamination by bad channels. The robust referencing approach produces the same results as average referencing if there are no bad channels”. However, “researchers should proceed with caution when there are not enough channels to cover the head for accurate channel interpolation” (Bigdely-Shamlo et al., 2015).

#### Electrode Positions Digitizer: Xensor^TM^

Before each EEG recording, we digitized the 3D locations of all electrodes and three major fiducials (nasion, left and right preauricular points) using the optical ANT Neuro xensor^TM^ system (ANT Neuro, Enschede, Netherlands^3^). Thus, we collected an individual electrodes digitization for each EEG recording.

### Interpolation of Electrode Positions

Positions of electrodes did not overlap for all the different EEG systems. For example, asalab^TM^ had over a hundred electrodes, and Emotiv EPOC had only fourteen. Additionally, actiCAP had a spherical montage but Emotiv EPOC and g.Nautilus used the 10-20 international system, and asalab^TM^ used the 10-5 international system. The different electrode locations did not allow us to directly compare activity from electrodes in the same places across the different EEG systems. Moreover, subjects had different head sizes, but not all systems had multiple cap sizes. Emotiv EPOC and g.Nautilus had only one cap size (these models had wires covered in a resilient plastic). This could have resulted in possibly different brain areas under the electrodes from different EEG systems. Additionally, for the same subject and the same EEG system, there could have been shifts of a few centimeters across data collection sessions due to a bias in the placement procedure of the cap.

To overcome these issues, we interpolated channel positions (and their activities) over a mesh-head model, taken from EEGLAB (colin27headmesh.mat) that consisted of 1082 mesh points. After the interpolation (EEGLAB function *headplot.m*), we calculated the activity of 1082 mesh channels. Mesh points are depicted as white dots on the head model in Figure 3A. Black labels represent the 10-20 international system and depict positions of 35 electrodes. The procedure for each recording session consisted of two steps: (1) The first step is to project positions of electrodes digitized with xensor^TM^ onto a mesh-head model. This step was necessary because the mesh-head model and subjects’ head forms were not exactly the same. (2) The second step was to interpolate electrode positions and activity of related channels over the positions of 1082 mesh points (mesh channels). After that, the 1082 mesh channels had the same positions on the mesh-head model in all EEG recordings in the study. Therefore, we could compare selected mesh channels between all EEG recordings in the study.

For each paradigm we found a region of interest and selected a cluster of interpolated mesh channels (see definition of clusters for the paradigms below in the results). Due to the varying density of electrodes across EEG systems, some clusters in a part of recordings may lack of electrodes within the clusters. Table 2 demonstrates average distances from clusters to nearest electrodes.

### Statistical Analysis

#### ANOVA

Repeated measurements of a paradigm recorded with different subjects, systems, or in different sessions encompass some variance related to these factors. We wanted to know to what extent each of these factors influenced the variance. The analysis of variance (ANOVA) approach offers itself to statistically estimate which factor influenced EEG data the most. Therefore, we used a three-way ANOVA of factors: System, Subject and Session and their two-factor interactions (Matlab function anovan.m, Sum of Squares (SS) Type III). ANOVA is from the family of linear models, and allows to model which of the different factors (Subject, System, Session) explains how much of the variance. The SS value served as a measure of variance explained by the factor. ANOVA is a rather general approach which should be used before a more specific modeling is done.

We wanted to know whether repeated recordings introduced some variability. Therefore, we split a paradigm in each EEG recording into eight equal parts and analyzed each part individually. The specific number eight was a trade-off between a larger number of parts we needed and the length of an individual part, which is a function of the amount of data available. We estimated that eight parts were a reasonable compromise. The division into eight parts was not used to increase the number of available data, but only to have an estimate of the variance within one paradigm in a recording. Thus, we gained eight measurements of a dependent variable per paradigm and EEG recording. As we had 48 EEG recordings, we yielded 384 such measurements per paradigm for the statistical analysis ANOVA.

We did not want to be dependent on one specific paradigm. Therefore, we selected a whole range of paradigms frequently used in the literature and which covered a range of cognitive processes. We treated each paradigm itself like an own experiment and analyzed the sources of variance independently from all the others paradigms. Therefore, there is no interaction between ANOVAs for different paradigms.

**Figure 4:**
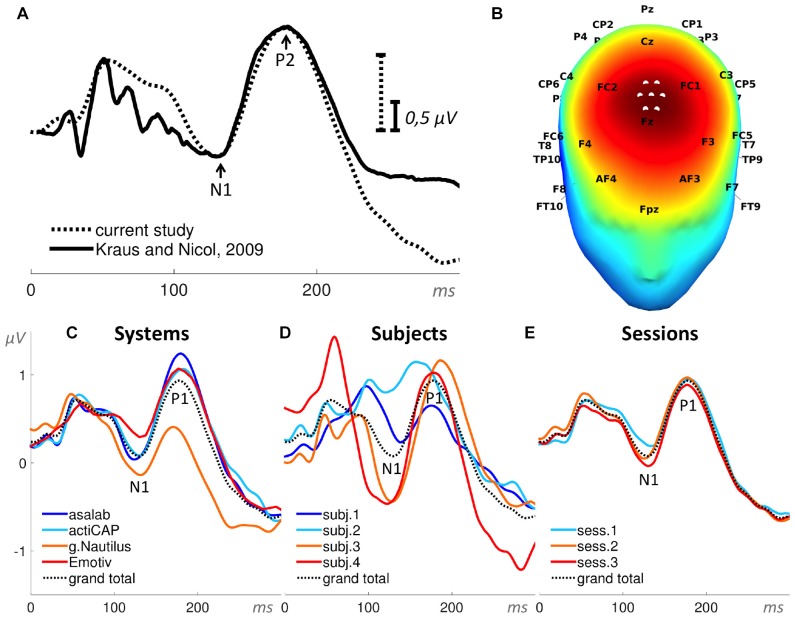
**Auditory evoked potential (AEP). (A)** Superposition of an event-related potential (ERP) from the literature (Kraus and Nicol, 2009) and the grand total ERP of all 48 sessions in the current study. We shifted the ERP from the literature by 16 ms to the right and reduced the voltage scale by 2.66 times, in order to find the best superposition of these two ERPs. **(B)** 3D map of the grand total ERP in the current study at 176 ms after the stimulus onset (P2 component in panel **A**). Seven white dots on the top of the head depict the cluster of mesh channels selected for further analysis. The central point of the cluster was [ 0 37 85 ] (Montreal Neurological Institute, MNI coordinates), the radius of the cluster was 20 mm. Black labels represent the 10-20 international system. **(C–E)** Grand average ERPs of the factors specified in the title above each panel and derived from the cluster of selected mesh channels (shown in panel **B**). Each ERP (colored lines) in panels **(C,D)** is based on 12 sessions grouped by a level of a factor and in panel **(E)** on 16 sessions, e.g., the dark blue ERP in panel **(C)** represents the grand average ERP of 12 sessions recorded with the asalab^TM^ EEG system and with four subjects, each recorded three times. The black dotted ERP in each panel is the grand total ERP of all 48 sessions in the study.

**Figure 5:**
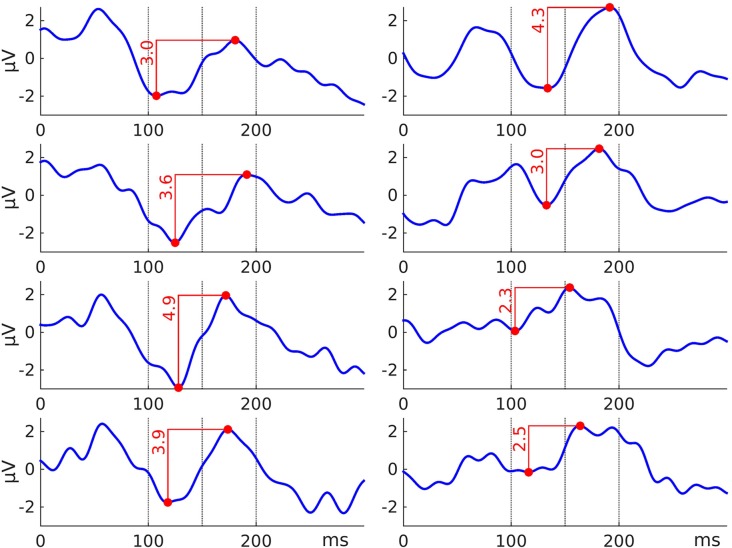
**Eight ERPs yielded from eight sequential parts of a recording session (in the figure: asalab^TM^, subject 4, session 3).** Each ERP derived from 119 trials. Vertical dotted lines indicate intervals used for finding minimum (N1) and maximum (P2) peaks in the paradigm on intervals 100–150 ms and 150–200 ms after a stimulus onset, respectively. The red numbers indicate peak-to-peak amplitudes (N1—P2) for each part. We processed 48 recordings and yielded 384 such peak-to-peak amplitude values of the paradigm.

In Paradigm 1 we measured for each part (119 trials per part) a peak-to-peak amplitude N1—P2 (Figures 4, 5). The red numbers in Figure 5 indicate peak-to-peak amplitudes N1—P2 for each part. The search interval for the peaks was from 100 ms to 200 ms relative to a stimulus onset.

**Table 2:** **Average distances between a cluster of selected mesh channels in a paradigm and the nearest electrode to the cluster in a recording session**.

	AEPs	SSVEP	MPs	vMMN	N170	vDM
asalab (127 chan.)	0.0	0.0	0.0	0.0	0.0	0.0
actiCAP (64 chan.)	0.4	0.0	0.3	0.0	0.1	0.1
g.Nautilus (32 chan.)	1.3	0.0	0.7	0.3	0.7	0.0
Emotiv EPOC (14 chan.)	1.4	2.4	0.5	1.0	1.3	0.8

**Figure 6:**
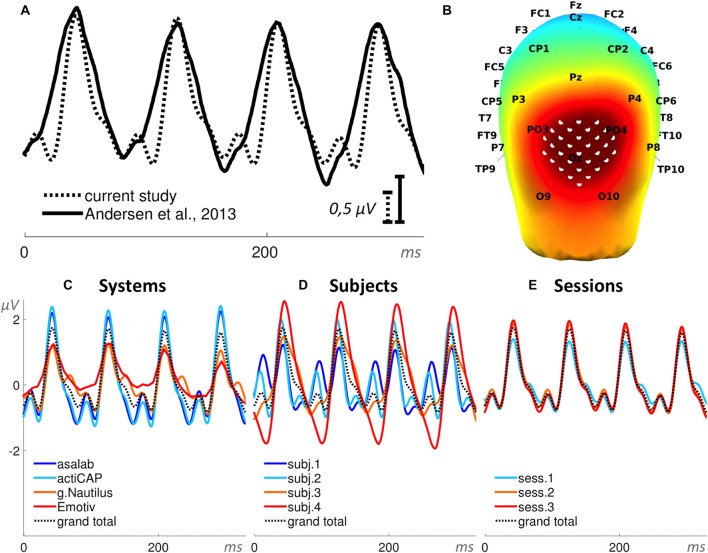
**Steady-state visually evoked potential (SSVEP). (A)** Superposition of an ERP from the literature (Andersen et al., 2013) and the grand total ERP of all 48 sessions in the current study. We shifted the ERP from the literature by 40 ms to the left and increased the voltage scale by 1.54 times, in order to find the best superposition of these two ERPs.** (B)** 3D map of the grand total ERP in the current study at 126 ms after the stimulus onset (second positive peak in panel **A**). Thirty-one white dots on the back of the head depict the cluster of mesh channels selected for further analysis. The central point of the cluster is [ 0 −118 14 ] (MNI coordinates), the radius of the cluster is 40 mm. Black labels represent the 10-20 international system. **(C–E)** Grand average ERPs of the factors specified in the title above each panel and derived from the cluster of selected mesh channels (shown in panel **B**). Each ERP (colored lines) in panels **(C,D)** is based on 12 sessions grouped by a level of a factor and in panel **(E)** on 16 sessions, e.g., the dark blue ERP in panel **(C)** represents the grand average ERP of 12 sessions recorded with the asalab^TM^ EEG system and with four subjects, each recorded three times. The black dotted ERP in each panel is the grand total ERP of all 48 sessions in the study.

In Paradigm 2 we measured for each part (270 trials per part) a peak-to-peak amplitude (minimum-to-maximum) of a SSVEP (Figure 6). The search interval for the peaks was from 120 ms to 220 ms relative to a stimulus onset.

**Figure 7:**
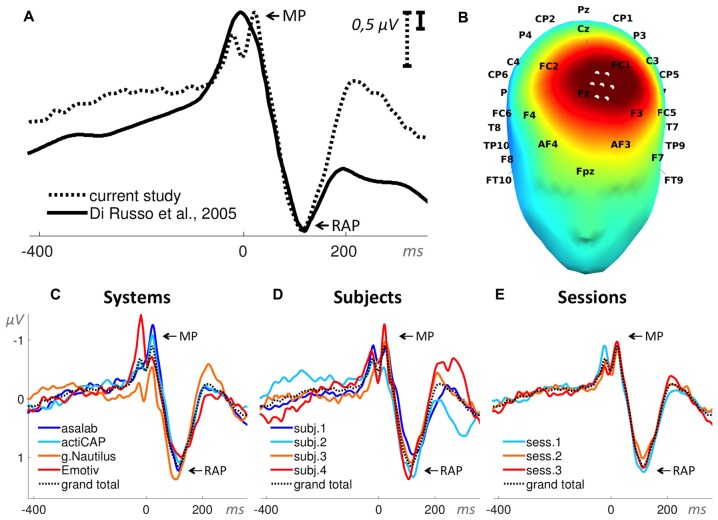
**Motor potentials (MPs). (A)** Superposition of an ERP from the literature (Di Russo et al., 2005) and the grand total ERP of all 48 sessions in the current study. We shifted the ERP from the literature by 53 ms to the left and reduced the voltage scale by 3.3 times, in order to find the best superposition of these two ERPs. The peak-to-peak amplitude of the ERP components MP and Reafferent Potential (RAP) defined the dependent variable in this paradigm. **(B)** 3D map of the grand total ERP in the current study at 115 ms after the stimulus onset (RAP component in panel **A**). Seven white dots on the front top of the head depict the cluster of mesh channels selected for further analysis. The central point of the cluster is [ −16 48 76 ] (MNI coordinates), the radius of the cluster is 20 mm. Black labels represent the 10-20 international system. **(C–E)** Grand average ERPs of the factors specified in the title above each panel and derived from the cluster of selected mesh channels (shown in panel **B**). Each ERP (colored lines) in panels **(C,D)** is based on 12 sessions grouped by a level of a factor and in panel **(E)** on 16 sessions, e.g., the dark blue ERP in panel **(C)** represents the grand average ERP of 12 sessions recorded with the asalab^TM^ EEG system and with four subjects, each recorded three times. The black dotted ERP in each panel is the grand total ERP of all 48 sessions in the study.

In Paradigm 3 we measured for each part (85 trials per part) a peak-to-peak amplitude MP—RAP (Figure 7). The search interval for the peaks was −40 ms to 140 ms relative to a stimulus onset.

**Figure 8:**
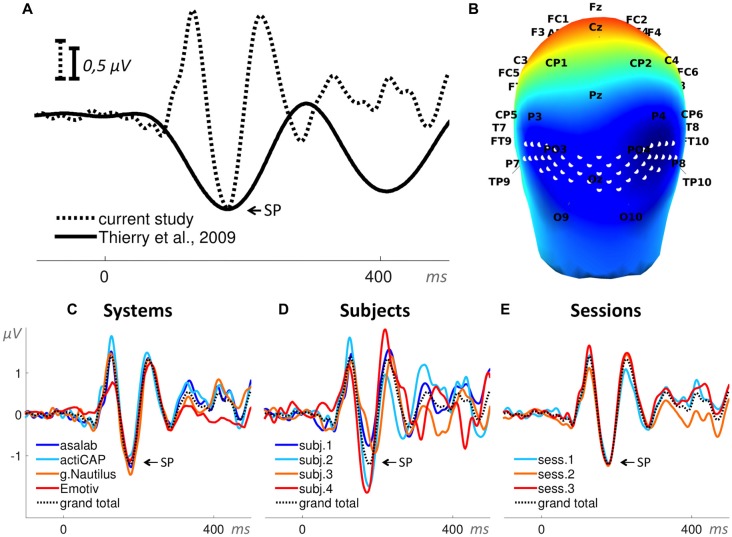
**Visual mismatch negativity (vMMN; ERP waveforms subtraction “deviant minus standard”). (A)** Superposition of an ERP from the literature (Thierry et al., 2009) and the grand total ERP (subtraction “deviant minus standard”) of all 48 sessions in the current study. We shifted the ERP from the literature by 10 ms to the left and reduced the voltage scale by 1.25 times, in order to find the best superposition of these two ERPs. **(B)** 3D map of the grand total ERP (subtraction “deviant minus standard”) in the current study at 180 ms after the stimulus onset (subtraction peak (SP) in panel **A**). Forty-eight white dots on the back of the head depict the cluster of mesh channels selected for further analysis. The central point of the cluster is [ 0 −118 14 ] (MNI coordinates). Black labels represent the 10-20 international system. **(C–E)** Grand average ERPs of the factors specified in the title above each panel and derived from the cluster of selected mesh channels (shown in panel **B**). Each ERP (colored lines) in panels **(C,D)** is based on 12 sessions grouped by a level of a factor and in panel **(E)** on 16 sessions, e.g., the dark blue ERP in panel **(C)** represents the grand average ERP (subtraction “deviant minus standard”) of 12 sessions recorded with the asalab^TM^ EEG system and with four subjects, each recorded three times. The black dotted ERP in each panel is the grand total ERP (subtraction “deviant minus standard”) of all 48 sessions in the study.

In Paradigm 4 we derived two ERPs from each part: deviant stimuli ERP (25 trials per part) and standard stimuli ERP (100 trials per part). We measured a negative peak amplitude of a resulting subtraction of deviant stimuli ERP minus standard stimuli ERP for each part (Figure 8). The search interval for the negative peak was 150–220 ms relative to a stimulus onset. We used the negative peak amplitude as the dependent variable.

**Figure 9:**
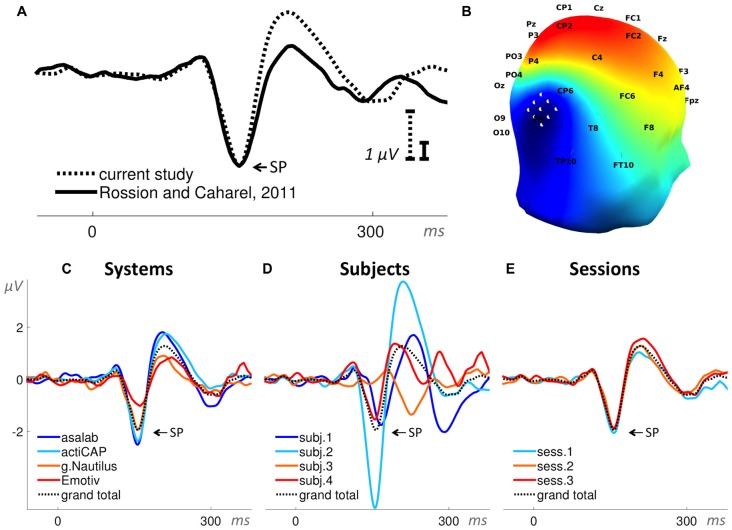
**Face-sensitive N170 component (ERP waveforms subtraction “faces minus cars”). (A)** Superposition of an ERP from the literature (Rossion and Caharel, 2011) and the grand total ERP (subtraction “faces minus cars”) of all 48 sessions in the current study. We shifted the ERP from the literature by 23 ms to the right and reduced the voltage scale by 2.95 times, in order to find the best superposition of these two ERPs. **(B)** 3D map of the grand total ERP (subtraction: face minus car) in the current study at 160 ms after the stimulus onset (subtraction peak (SP) in panel **A**). Nine white dots on the right side of the head depict the cluster of mesh channels selected for further analysis. The central point of the cluster is [ 70 −83 6 ] (MNI coordinates), the radius of the cluster is 22 mm. Black labels represent the 10-20 international system. **(C–E)** Grand average ERPs of the factors specified in the title above each panel and derived from the cluster of selected mesh channels (shown in panel **B**). Each ERP (colored lines) in panels **(C,D)** is based on 12 sessions grouped by a level of a factor and in panel **(E)** on 16 sessions, e.g., the dark blue ERP in panel **(C)** represents the grand average ERP (subtraction “faces minus cars”) of 12 sessions recorded with the asalab^TM^ EEG system and with four subjects, each recorded three times. The black dotted ERP in each panel is the grand total ERP (subtraction “faces minus cars”) of all 48 sessions in the study.

In Paradigm 5 we derived two ERPs from each part: face stimuli ERP (40 trials per part) and car stimuli ERP (40 trials per part). We measured a negative peak amplitude of a resulting subtraction of face stimuli ERP minus car stimuli ERP for each part (Figure 9). The search interval for the negative peak was 130–190 ms relative to a stimulus onset. We used the negative peak amplitude as the dependent variable.

**Figure 10:**
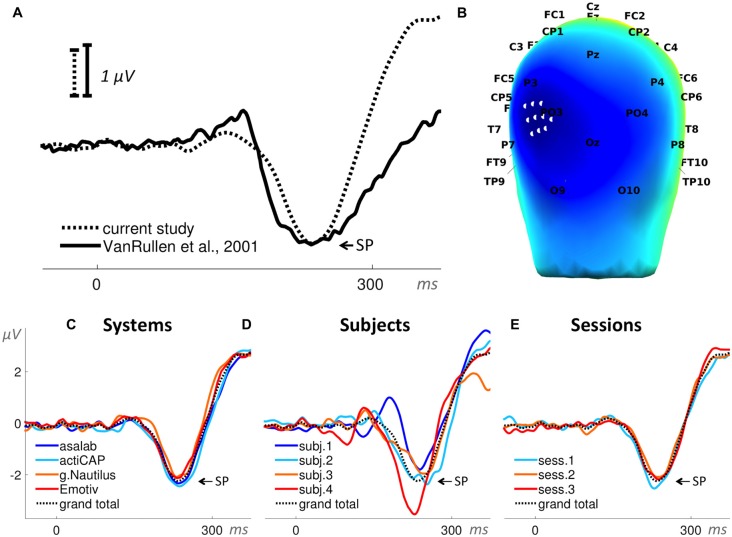
**Visual decision-making (vDM) N240 component (ERP waveforms subtraction “targets minus distractors”). (A)** Superposition of an ERP from the literature (VanRullen and Thorpe, 2001) and the grand total ERP (subtraction “targets minus distractors”) of all 48 sessions in the current study. We shifted the ERP from the literature by 23 ms to the right and increased the voltage scale by 1.11 times, in order to find the best superposition of these two ERPs. **(B)** 3D map of the grand total ERP (subtraction “targets minus distractors”) in the current study at 235 ms after the stimulus onset (subtraction peak (SP) in panel **A**). Ten white dots on the left back side of the head depict the cluster of mesh channels selected for further analysis. The central point of the cluster is [−58 −90 28] (MNI coordinates), the radius of the cluster is 20 mm. Black labels represent the 10-20 international system. **(C–E)** Grand average ERPs of the factors specified in the title above each panel and derived from the cluster of selected mesh channels (shown in panel **B**). Each ERP (colored lines) in panels **(C,D)** is based on 12 sessions grouped by a level of a factor and in panel **(E)** on 16 sessions, e.g., the dark blue ERP in panel **(C)** represents the grand average ERP (subtraction “targets minus distractors”) of 12 sessions recorded with the asalab^TM^ EEG system and with four subjects, each recorded three times. The black dotted ERP in each panel is the grand total ERP (subtraction “targets minus distractors”) of all 48 sessions in the study.

In Paradigm 6 we derived two ERPs from each part: target stimuli ERP (25 trials per part) and distractor stimuli ERP (80 trials per part). We measured a negative peak amplitude of a resulting subtraction of target stimuli ERP minus distractor stimuli ERP for each part (Figure 10). The search interval for the negative peak was 200–280 ms relative to a stimulus onset. We used the negative peak amplitude as the dependent variable.

Despite the differences in sampling rates of the four EEG systems, interpolation to a common sampling frequency was not required, since we used search intervals to measure amplitude peaks. However, to build grand totals in Figures 4, 6–10, we did interpolation to a common sampling frequency of 1000 Hz for all systems.

#### *Post hoc* Test

To estimate which levels of a factor were significantly different from each other and therefore explain the variance within the factor, we conducted the *post hoc* multiple comparison test (Matlab function *multcompare.m*).

## Results

### Paradigms

#### Paradigm 1: Auditory Evoked Potentials

Late-latency AEPs “beginning with P1 (which is sometimes classified as middle-latency) at about 80 ms through to N2 at about 250 ms, all are cortical in origin and maximal in amplitude at the central top of the scalp” (Kraus and Nicol, 2009). The 3D map in Figure 4B demonstrates the scalp distribution of the grand total ERP at 176 ms after the stimulus onset (P2 component) in the current study. Seven white dots on the top of the head depict the cluster of mesh channels selected for further analysis. The central point of the cluster is [ 0 37 85 ] in Montreal Neurological Institute (MNI) coordinates, the radius of the cluster is 20 mm. All further results in this paradigm were based on the average activity of the selected mesh channels of the cluster. In order to nullify possible influences of baseline position on P2 amplitude value, we selected the peak-to-peak amplitude of the components N1 and P2 as a dependent variable in the paradigm.

A comparison of ERPs taken from scientific literature and the current study is shown in Figure 4A, which demonstrates similarity of these ERPs. Early auditory components of the ERP in the current study were smoothed, however, the most prominent components N1 and P2 have nearly identical forms. Grand average ERPs per level of a factor in Figures 4C–E reveal that the peak-to-peak amplitude of the components N1 and P2 vary the most by the factor Subject, less by the factor System, and vary the least by the factor Session.

**Figure 11:**
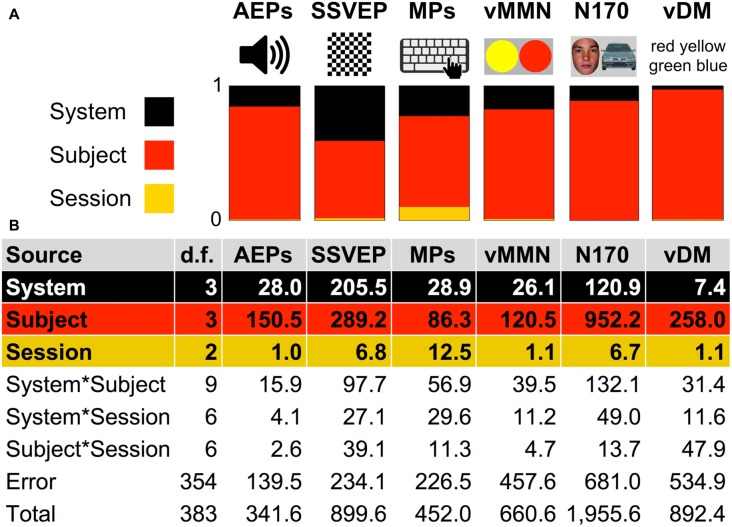
**Analysis of variance (ANOVA) Sum of Squares (SS) summary of six paradigms. (A)** Color bars represent ratios of SS of factors: System, Subject and Session in the paradigms AEPs, SSVEP, MPs, vMMN, N170, and vDM. **(B)** ANOVA table summary of SS due to each source for the six paradigms. “d.f.”: degrees of freedom associated with each source.

The ANOVA results found statistical significance for these two factors: System (SS = 28.0, *F* = 23.7, *p* < 0.01) and Subject (SS = 150.5, *F* = 127.3, *p* < 0.01). The factor Session was not statistically significant in the ANOVA results (SS = 1.0, *F* = 1.3, *p* = 0.29). One two-factor interaction was statistically significant: System*Subject (SS = 15.9, *F* = 1.8, *p* < 0.01). Values of SS_Error_ and SS_Total_ were equal to 139.5 and 341.6 respectively. The ratio of SS_System_, SS_Subject_ and SS_Session_ was 16%, 83%, and 1%, respectively (Figure 11). ANOVA results in the paradigm revealed that the factor Subject was the biggest source of variance, relative to other factors.

See *post hoc*-test summary for all paradigms in Figure 13. In the factor System (Figure 13A) with four levels (asalab^TM^, actiCAP, g.Nautilus, Emotiv), asalab^TM^’s mean value was significantly different from g.Nautilus’s and Emotiv’s mean values; actiCAP’s mean value was significantly different from g.Nautilus’s mean value; g.Nautilus’s mean value was significantly different from asalab^TM^’s, actiCAP’s, and Emotiv’s mean values; Emotiv’s mean value was significantly different from g.Nautilus’s and asalab^TM^’s mean values. In the factor Subject (Figure 13B) with four levels (subject 1, subject 2, subject 3, subject 4), subject 1’s mean value was significantly different from the mean values of subjects 3 and 4; subject 2’s mean value was significantly different from the mean values of subjects 3 and 4; subject 3’s mean value was significantly different from the mean values of subjects 1 and 2; subject 4’s mean value was significantly different from the mean values of subjects 1 and 2. In the factor Session (Figure 13C) with three levels (session 1, session 2, session 3), mean values of all three levels of the factor were not significantly different from each other. The *post hoc* test revealed (Figure 13) that two research-grade EEG systems (asalab^TM^ and actiCAP) demonstrated similar results to each other. Emotiv and g.Nautilus demonstrated significantly different results from one or both of the research-grade EEG systems, respectively. Subjects split up in two statistically distinguishable groups: the first group—subjects 1 and 2 and the second group—subjects 3 and 4.

**Figure 12:**
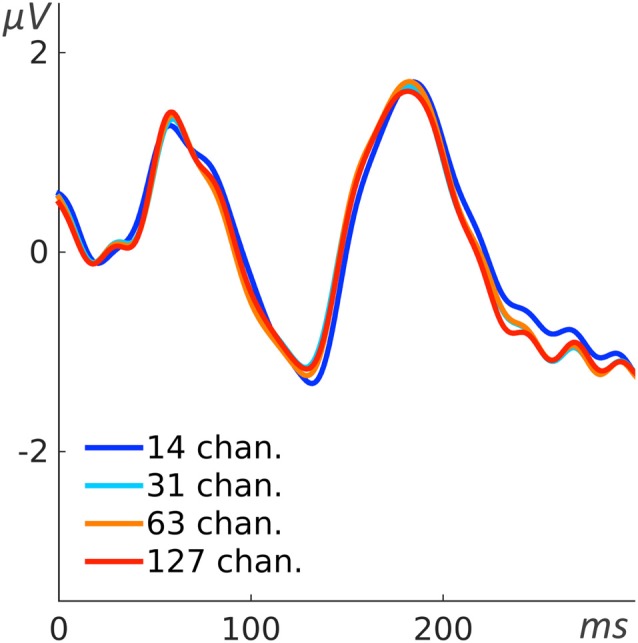
**Interpolation-related error.** Four ERPs gained from the AEP paradigm. The red line indicates the original data of one recording session of one subject with the 127 channel system (asalab). The additional lines indicate the evoked potential obtained by downsampling the number of channels to 63 (orange), 31 (light blue) and 14 (dark blue), respectively and interpolating to 1082 mesh-head channels.

**Figure 13:**
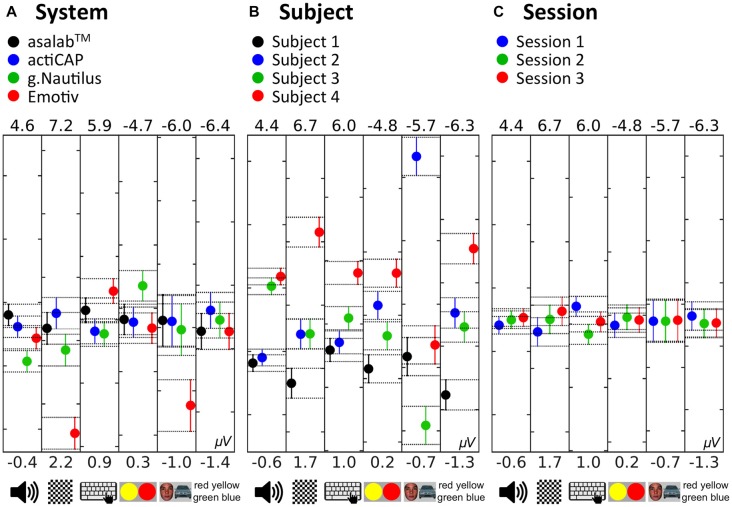
***Post hoc* test: “multiple comparison of means” in the six paradigms. (A)** Comparison of levels of the factor System. **(B)** Comparison of levels of the factor Subject. **(C)** Comparison of levels of the factor Session. Colored dots in the plots show the mean value of the level of the factor, e.g., the black dot in the first plot from the left is equal to 1.8 μV and represents the mean value of 96 measurements of the dependent variable (12 EEG recordings × 8 measurements per recording) in the paradigm AEPs with the asalab^TM^ EEG system. Error bars and related horizontal dotted lines indicate comparison intervals. If two dots have intersecting comparison intervals, then they are not significantly different, otherwise they are. Values above and below each plot show *Y*-axis limits of the plot. Additionally, all plots have 5 μV interval between the lower and upper *Y*-axis limits. The first tree plots in each panel represent paradigms which investigate a peak-to-peak amplitude of an ERP, where a higher positive value correlates with a larger manifestation of an effect, therefore these plots have the normal direction of *Y*-axis. The last three plots in each panel represent paradigms which investigate amplitude of a subtraction peak (SP) of two ERP waveforms, where a higher negative value correlates with a larger manifestation of an effect, therefore these plots have the reverse direction of *Y*-axis. Thus, the higher a dot in all of these plots is located, the larger the manifestation of the effect in a paradigm is. Pictograms on the bottom of each plot show the paradigm related to the plot.

#### Paradigm 2: Steady-State Visually Evoked Potential (SSVEP)

The ANOVA results found statistical significance for all three factors: System (SS = 205.5, *F* = 103.6, *p* < 0.01), Subject (SS = 289.2, *F* = 145.8, *p* < 0.01), and Session (SS = 6.8, *F* = 5.1, *p* < 0.01). All three two-factor interactions were statistically significant: System*Subject (SS = 97.7, *F* = 16.4, *p* < 0.01), System*Session (SS = 27.1, *F* = 6.8, *p* < 0.01), and Subject*Session (SS = 39.1, *F* = 9.8, *p* < 0.01). Values of SS_Error_ and SS_Total_ were equal to 234.1 and 899.6 respectively. The ratio of SS_System_, SS_Subject_, and SS_Session_ was 41%, 58%, and 1%, respectively (Figure 11). ANOVA results in the paradigm revealed that the factor Subject was the biggest source of variance, relative to other factors.

See *post hoc*-test summary for all paradigms in Figure 13. In the factor System (Figure 13A) with four levels (asalab^TM^, actiCAP, g.Nautilus, Emotiv), asalab^TM^’s mean value was significantly different from Emotiv’s mean value; actiCAP’s mean value was significantly different from g.Nautilus’s and Emotiv’s mean values; g.Nautilus’s mean value was significantly different from actiCAP’s and Emotiv’s mean values; Emotiv’s mean value was significantly different from asalab^TM^’s, actiCAP’s, and g.Nautilus’s mean values. In the factor Subject (Figure 13B) with four levels (subject 1, subject 2, subject 3, subject 4), subject 1’s mean value was significantly different from the mean values of subjects 2, 3, and 4; subject 2’s mean value was significantly different from the mean values of subjects 1 and 4; subject 3’s mean value was significantly different from the mean values of subjects 1 and 4; subject 4’s mean value was significantly different from the mean values of subjects 1, 2 and 3. In the factor Session (Figure 13C) with three levels (session 1, session 2, session 3), mean values of all three levels of the factor were not significantly different from each other. The *post hoc* test revealed (Figure 13) that two research-grade EEG systems (asalab^TM^ and actiCAP) demonstrated similar results to each other. g.Nautilus and Emotiv demonstrated significantly different results from one or both of the research-grade EEG systems, respectively. Subjects split up in three statistically distinguishable groups: the first group—subject 1, the second group—subjects 2 and 3, and the third group—subject 4.

#### Paradigm 3: Motor Potentials (MPs) Elicited by Voluntary Tapping

The ANOVA results found statistical significance for all three factors: System (SS = 28.9, *F* = 15.1, *p* < 0.01), Subject (SS = 86.3, *F* = 45.0, *p* < 0.01), and Session (SS = 12.5, *F* = 9.8, *p* < 0.01). All three two-factor interactions were statistically significant: System*Subject (SS = 56.9, *F* = 9.9, *p* < 0.01), System*Session (SS = 29.6, *F* = 7.7, *p* < 0.01), and Subject*Session (SS = 11.3, *F* = 2.9, *p* < 0.01). Values of SS_Error_ and SS_Total_ were equal to 226.5 and 452.0 respectively. The ratio of SS_System_, SS_Subject_, and SS_Session_ was 23%, 67%, and 10%, respectively (Figure 11). ANOVA results in the paradigm revealed that the factor Subject was the biggest source of variance, relative to other factors.

See *post hoc*-test summary for all paradigms in Figure 13. In the factor System (Figure 13A) with four levels (asalab^TM^, actiCAP, g.Nautilus, Emotiv), asalab^TM^’s mean value was not significantly different from mean values of the three other EEG systems; actiCAP’s mean value was significantly different from Emotiv’s mean value; g.Nautilus’s mean value was significantly different from Emotiv’s mean value; Emotiv’s mean value was significantly different from actiCAP’s and g.Nautilus’s mean values. In the factor Subject (Figure 13B) with four levels (subject 1, subject 2, subject 3, subject 4), subject 1’s mean value was significantly different from the mean values of subjects 3 and 4; subject 2’s mean value was significantly different from the mean values of subjects 3 and 4; subject 3’s mean value was significantly different from the mean values of subjects 1, 2, and 4; subject 4’s mean value was significantly different from the mean values of subjects 1, 2 and 3. In the factor Session (Figure 13C) with three levels (session 1, session 2, session 3), session 1’s mean value was significantly different from session 2’s mean value; session 2’s mean value was significantly different from session 1’s mean value; session 3’s mean value was not significantly different from mean values of the other two sessions. The *post hoc* test revealed (Figure 13) that two research-grade EEG systems (asalab^TM^ and actiCAP) demonstrated similar results to each other. Emotiv demonstrated significantly different results from one of the research-grade EEG system. Subjects split up in three statistically distinguishable groups: the first group—subjects 1 and 2, the second group—subject 3, and the third group—subject 4.

#### Paradigm 4: Visual Mismatch Negativity (vMMN)

The ANOVA results found statistical significance for these two factors: System (SS = 26.1, *F* = 6.7, *p* < 0.01) and Subject (SS = 120.5, *F* = 31.1, *p* < 0.01). The factor Session was not statistically significant (SS = 1.1, *F* = 0.4, *p* = 0.66). One two-factor interaction was statistically significant: System*Subject (SS = 39.5, *F* = 3.4, *p* < 0.01). Values of SS_Error_ and SS_Total_ were equal to 457.6 and 660.6 respectively. The ratio of SS_System_, SS_Subject_, and SS_Session_ was 18%, 81%, and 1%, respectively (Figure 11). ANOVA results in the paradigm revealed that the factor Subject was the biggest source of variance, relative to other factors.

See *post hoc*-test summary for all paradigms in Figure 13. In the factor System (Figure 13A) with four levels (asalab^TM^, actiCAP, g.Nautilus, Emotiv), asalab^TM^’s mean value was significantly different from g.Nautilus’s mean value; actiCAP’s mean value was significantly different from g.Nautilus’s mean value; g.Nautilus’s mean value was significantly different from asalab^TM^’s, actiCAP’s and Emotiv’s mean values; Emotiv’s mean value was significantly different from g.Nautilus’s mean value. In the factor Subject (Figure 13B) with four levels (subject 1, subject 2, subject 3, subject 4), mean values of all four levels of the factor were significantly different from each other. In the factor Session (Figure 13C) with three levels (session 1, session 2, session 3), mean values of all three levels of the factor were not significantly different from each other. The *post hoc* test revealed (Figure 13) that two research-grade EEG systems (asalab^TM^ and actiCAP) demonstrated similar results to each other. g.Nautilus demonstrated significantly different results from both of the research-grade EEG systems. All four subjects’ had significantly different from each other mean values.

#### Paradigm 5: Face-Sensitive N170 Component

The ANOVA results found statistical significance for these two factors: System (SS = 120.9, *F* = 21.0, *p* < 0.01) and Subject (SS = 952.2, *F* = 165.0, *p* < 0.01). The factor Session was not statistically significant (SS = 6.7, *F* = 1.7, *p* = 0.18). Two two-factor interactions were statistically significant: System*Subject (SS = 132.1, *F* = 7.6, *p* < 0.01) and System*Session (SS = 49.0, *F* = 4.2, *p* < 0.01). Values of SS_Error_ and SS_Total_ were equal to 681.0 and 1955.6 respectively. The ratio of SS_System_, SS_Subject_, and SS_Session_ was 11%, 88%, and 1%, respectively (Figure 11). ANOVA results in the paradigm revealed that the factor Subject was the biggest source of variance, relative to other factors.

See *post hoc*-test summary for all paradigms in Figure 13. In the factor System (Figure 13A) with four levels (asalab^TM^, actiCAP, g.Nautilus, Emotiv), asalab^TM^’s mean value was significantly different from Emotiv’s mean value; actiCAP’s mean value was significantly different from Emotiv’s mean value; g.Nautilus’s mean value was significantly different from Emotiv’s mean value; Emotiv’s mean value was significantly different from asalab^TM^’s, actiCAP’s, and g.Nautilus’s mean values. In the factor Subject (Figure 13B) with four levels (subject 1, subject 2, subject 3, subject 4), subject 1’s mean value was significantly different from the mean values of subjects 2 and 3; subject 2’s mean value was significantly different from the mean values of subjects 1, 3, and 4; subject 3’s mean value was significantly different from the mean values of subjects 1, 2, and 4; subject 4’s mean value was significantly different from the mean values of subjects 2 and 3. In the factor Session (Figure 13C) with three levels (session 1, session 2, session 3), mean values of all three levels of the factor were not significantly different from each other. The *post hoc* test revealed (Figure 13) that two research-grade EEG systems (asalab^TM^ and actiCAP) demonstrated similar results to each other. Emotiv demonstrated significantly different results from both of the research-grade EEG systems. Subjects split up in three statistically distinguishable groups: the first group—subjects 1 and 4, the second group—subject 2, and the third group—subject 3.

#### Paradigm 6: Visual Decision-Making N240 Component (vDM)

The ANOVA results found statistical significance for the following factor: Subject (SS = 258.0, *F* = 56.9, *p* < 0.01). The factors System (SS = 7.4, *F* = 1.6, *p* = 0.18) and Session (SS = 1.1, *F* = 0.4, *p* = 0.69) were not statistically significant. One two-factor interaction was statistically significant: Subject*Session (SS = 47.9, *F* = 5.3, *p* < 0.01). Values of SS_Error_ and SS_Total_ were equal to 534.9 and 892.4 respectively. The ratio of SS_System_, SS_Subject_, and SS_Session_ was 3%, 97%, and 0%, respectively (Figure 11). ANOVA results in the paradigm revealed that the factor Subject was the biggest source of variance, relative to other factors.

See *post hoc*-test summary for all paradigms in Figure 13. In the factor System (Figure 13A) with four levels (asalab^TM^, actiCAP, g.Nautilus, Emotiv), mean values of all four levels of the factor were not significantly different from each other. In the factor Subject (Figure 13B) with four levels (subject 1, subject 2, subject 3, subject 4), subject 1’s mean value was significantly different from the mean values of subjects 2, 3, and 4; subject 2’s mean value was significantly different from the mean values of subjects 1 and 4; subject 3’s mean value was significantly different from the mean values of subjects 1 and 4; subject 4’s mean value was significantly different from the mean values of subjects 1, 2 and 3. In the factor Session (Figure 13C) with three levels (session 1, session 2, session 3), mean values of all three levels of the factor were not significantly different from each other. The *post hoc* test revealed (Figure 13) that two research-grade EEG systems (asalab^TM^ and actiCAP) demonstrated similar results to each other. Subjects split up in three statistically distinguishable groups: the first group—subjects 1, the second group—subjects 2 and 3, and the third group—subject 4.

### Summary

#### ANOVA

The ANOVA results are consistent across the six paradigms and suggest that the factor Subject was the largest source of variance in the study. However, ratio of variation over subjects (Figure 11) was smallest in the “low-level” tasks like flickering checkerboard (Figure 11—SSVEP) and highest in the more cognitive-oriented tasks like decision-making task (Figure 11—vDM). The factor System was also a significant source of variance in all paradigms. The factor Session was a relatively small source of variance, and it had a significant *p*-value (*p* < 0.01) only in two paradigms (MPs and SSVEP) out of six.

#### Interpolation-Related Error

We introduced the interpolation step of the physically recorded channels to 1082 mesh channels in order to make the different systems comparable at a selected location. This does, however, raise a question regarding the validity of whether the interpolation step creates abnormal, unrealistic or other spurious data resulting in increased variance between the systems or poorer performance based solely on channel density. To address this, we compared an exemplar original recording by the full set of electrodes with a reduced and interpolated version thereof. Specifically, we used data from the system with the highest electrode count (asalab) and selected channels that were also present in the other systems, i.e., Emotiv EPOC, g.Nautilus, or similar electrodes (actiCAP had a spherical montage) to create an artificially sparse, but matching montage. Based on this sub-selection of electrodes, we interpolated the signals for the region of interest similar to had been performed for all previous analyses (Figure 4B). We chose the AEP paradigm as a relevant example, as not all systems have good coverage of the region of interest. Distances between the AEP cluster of mesh channels and the nearest electrodes were 0 cm for the original 127 channels and the 63- and 31-channels down sampling. This means that at least one physical electrode was present right in the region of interest and close to the mesh points. The distance for the 14-channels down sampling was 3.2 cm, which is more than two times greater than the average distance for Emotiv EPOC (1.4 cm, see Table 2).

Figure 12 compares the AEP of the original recording and reduced, interpolated data sets. The high similarity of the evoked potentials demonstrates that here the interpolation step does not introduce relevant additional variance (Figure 12). Thus, in this case, as the topographic distribution of the evoked potential was relatively smooth, the interpolation could capture the evoked potential even though the distance of physical electrodes to the region of interest was relatively large. However, this does not imply that the number of electrodes is irrelevant. It only shows that the interpolation step does not by itself degrade the data or create artifactual results. The coverage of a system should be suited for a specific experiment.

#### *Post hoc* Test

The *post hoc* tests revealed that the two research-grade EEG systems (asalab^TM^ and actiCAP) had no significantly different values from each other in all paradigms (Figure 13). However, g.Nautilus had significantly different values from one or both of the research-grade EEG systems in 3 out of 6 paradigms, and Emotiv EPOC also had significantly different values from one or both of the research-grade EEG systems in 4 out of 6 paradigms. In 4 out of 6 paradigms, Emotiv EPOC showed a stronger deviation of values, than g.Nautilus. The *post hoc* test results also revealed subject differences. Subject 1 had the weakest effect responses (dependent variable) in 5 out of 6 paradigms. Subject 4 had the strongest effect responses (dependent variable) in 5 out of 6 paradigms. These results revealed subject specificity of EEG responses. The *post hoc* test results demonstrated that the factor Session influenced EEG results at the very least since, in 5 out of 6 paradigms, all 3 sessions were not significantly different from one another and only in the MPs paradigm sessions 1 and 2 were significantly different from each other. We did not observe any systematic increase or decrease of mean amplitude values in sequences of sessions (Figure 13C). *Post hoc* results were congruent with ANOVA results and suggested that the factor Subject was the largest source of variance in the study; the factor System was also the prominent source of variance, however, mostly because of the mobile EEG systems (g.Nautilus and Emotiv EPOC); and the factor Session was the smallest source or variance in the study.

## Discussion

The present results demonstrate that subjects are the largest source of variance of ERPs in all paradigms. On average, they explain 32% of the total variance. However, systems are also a significant source of variance in all paradigms, on average 9%. In contrast, sessions are a relatively small source of variance, on average 1%. However, this 1% does not mean that ERPs from two sequential recordings with the same EEG system and the same subject were nearly identical. Rather, we did not find any systematic bias of ERP amplitudes, based on the ordinal number of a recording for the same subject and the same system. The two standard research-grade EEG systems had no significantly different means from each other through all paradigms. However, the g.Nautilus with g.SAHARA electrodes and Emotiv EPOC EEG systems used in the current study demonstrated different mean values from one or both of the two standard research-grade EEG systems in at least half of the paradigms.

The variance across systems is smaller than variance across subjects but is not negligible. It is relatively easy to collect more subjects, average over subjects, and reduce the variance. Typically, the number of subjects per study ranges from 10 to 20. Our ANOVA evaluated the variance associated with different independent variables, e.g., Subject, System, and Session. This variance is independent of the number of subjects. If we would pool data from 16 subjects, that would improve the error of uncertainty in estimates of the mean (like ERP) by the square root of the number of subjects (sqrt(16)), thus resulting from current 32% to 8%. It is unusual to average across different systems as most studies do not have access to multiple EEG systems. The uncertainty due to EEG systems (9%) would be of the same order of magnitude as the uncertainty due to subjects (32%/sqrt(16) = 8%), if we would pool data from 16 subjects. Increasing the number of subjects even higher, e.g., to 100, would have progressively diminishing returns, because we are still left with the variance of the systems due to different laboratories using different EEG systems.

The variance across systems may appear due to variability in amplifiers, contact materials, number of electrodes, etc. However, it was not in the focus of the current study to disentangle these interactions and whether a system could be improved by tweaking an individual component. Instead, we assumed that manufacturers designed complete optimized packages, with different parts tailored suitably. Hence, we tested the complete systems as they were supplied by the manufacturer.

We used the most prominent ERPs in each paradigm to calculate the variance due to EEG systems. If we would focus on more subtle ERPs, like middle-latency AEPs, the variance related to systems could be higher. Modern EEG paradigms, which allow free movements under natural contexts, investigate prominent sources of EEG activity (Gwin et al., 2011), which are usually “cognitive” in origin and late latency in time (Sur and Sinha, 2009). BCI (Guger et al., 2012; Liu et al., 2012), gaming (Liao et al., 2012), and wellness (Dixit, 2011; Milazzo et al., 2013) use of EEG also rely on prominent sources of electrocortical activity. Investigation of subtle ERPs requires lab conditions with reduced number of noise factors, and advantages of mobile EEG systems are not relevant here. Therefore, it makes sense to compare prominent ERPs across EEG systems.

The variance across sessions, on average 1%, is the smallest source of variance. This 1% means that the systematic effect of knowing which session it is barely helps us to explain the variance. However, this does not mean that there is no variance across sessions. Indeed, adding up SS of the three main factors and their interactions do not add up to 100%. Therefore, the residual variance across sessions can substantially contribute to the Error SS term. In other words, the variance across sessions would be higher if, for example, subjects were always more anxious in the first than in the second session. Thus, the present results of the variance across sessions suggest that different sessions are interchangeable.

There are other means to compare EEG systems, for example, the correlation of waveforms from two different EEG systems during a simultaneous recording from electrodes located near each other (Gargiulo et al., 2010; Yeung et al., 2015). Many mobile EEG systems have a rigid montage, and it is not possible to overlay montages of two different EEG systems. Moreover, it is problematic to place two electrodes at the same spot, as well as the references. Potential differences can then be attributed either to the systems or to the different positions, which creates the problem. The interpolation approach presented in the current study can address this problem. The approach uses information from several physical electrodes to estimate the ERP at the optimal location as reported in the literature. It allows to have, first, the optimal ERP signal and second, a direct comparison of different EEG systems. To conduct the interpolation, we used standard algorithms supplied by EEGLAB. The interpolation approach allows us to compare virtual channels at same scalp positions directly. However, this approach should be used with caution when there are not enough channels to cover the head for accurate channel interpolation. Therefore, it might be the case that the differences observed for the Emotiv EPOC are still partly due to the lower coverage.

The sensitivity of a given EEG system for a given source inevitably depends on the location of the electrodes. Different systems are developed for different purposes and are not necessarily generic in their usage; certainly a high electrode count is a suitable way to ensure good coverage. For systems with a lower number of electrodes, their spatial distributions are not suitable for all experiments. Interpolating a moderate number of electrodes to a fine mesh might help in some situations. However, they lack flexibility as generic EEG systems. For the Emotiv EPOC especially the users should be aware of the shortcomings and use the system accordingly.

More subjects in the current study would be beneficial, as well as increasing the number of sessions with new montages. However, even with four subjects, four EEG systems, and three sessions with new montages, there are already 48 recording sessions. Therefore, a multicenter study involving a larger number of subjects and systems using the present benchmark is a desirable next step.

We compared different EEG systems in this study, but the ground truth of EEG signal remains unknown. For example, the two research-grade systems in the study produce similar results, but it is hard to argue whether these are closer to the unknown ground truth than others or not. There are studies which use a phantom head with controlled dipolar sources of electrical activity embedded in the phantom (Oliveira et al., 2016a). On the one side, such approach allows us to obtain results closer to the ground truth since the exact electrical stimulation of the phantom can be conducted on different EEG systems. On the other side of the phantom head approach, the real generators of EEG signal in the brain, as well as tissue conductivity and other less predictable properties of natural environment, are missed. While the ground truth of scalp EEG signals is unknown, the expectations from new mobile EEG systems are to produce comparable results to those of research-grade EEG systems, which they do to varying degree.

The mean amplitude values in Figure 13 are usually larger than they look on the ERPs in Figures 4, 6–10, panels **C–E**. To yield a measurement of a dependent variable, we used search intervals for each ERP of the eight sequential parts of a recording session (Figure 5), but the ERPs in Figures 4, 6–10 were yielded by a standard averaging procedure of ERPs from the eight sequential parts of a recording session. Therefore, these search intervals made the mean amplitude values in Figure 13 larger than they look on the ERPs in Figures 4, 6–10, panels **C–E**.

We encountered a time displacement of the grand total ERPs between the current study and respective examples from the literature. We think that the reason for that is that different EEG systems were used in the literature examples and in the current study. Indeed, we also encountered a time displacement of 11 ms in the current study between g.Nautilus and the research-grade EEG systems (asalab^TM^ and actiCAP). Such time displacements may happen due to a variety of technical reasons. The same problem of time alignment between EEG systems was also encountered in other studies (Gargiulo et al., 2010; Ries et al., 2014). However, these issues did not influence measurements of dependent variables in the study.

The current study proposes an approach to evaluate new mobile EEG systems by means of ERP responses. The developed benchmark of six paradigms and yielded results can be used for evaluation of new EEG systems. In contrast to other studies (Gargiulo et al., 2010; Guger et al., 2012; Liao et al., 2012; De Vos et al., 2014; Yeung et al., 2015; Oliveira et al., 2016b), which compare EEG signals from different systems, we match the difference between EEG systems to other factors of variance, like subjects and sessions, in order to estimate the importance of the difference between EEG systems. We also demonstrated the mesh-head-model interpolation approach, which addresses the issue of not overlapping EEG montages of different EEG systems. Results showed that research-grade EEG systems are indeed mature since they have no significantly different means through all paradigms between the two systems. The g.Nautilus with g.SAHARA electrodes and Emotiv EPOC EEG systems used in the current study are in many paradigms as good as current research-grade systems.

## Author Contributions

AM, SMK, WDH, DPF and PK conceived and designed the experiments; AM, PL and KI performed the experiments; AM and PK analyzed the data; AM, WDH, DPF and PK wrote and reviewed the article.

## Funding

This work was supported by the Cognition and Neuroergonomics Collaborative Technology Alliance (W911NF-10-2-0022).

## Conflict of Interest Statement

The authors declare that the research was conducted in the absence of any commercial or financial relationships that could be construed as a potential conflict of interest. The handling editor declared a past co-authorship with one of the authors PK and states that the process nevertheless met the standards of a fair and objective review.

## Footnotes

^1^www.ant-neuro.com

^2^www.brainproducts.com

^3^www.ant-neuro.com/products/xensor
